# A phase 1 dose-finding and pharmacokinetic study of acasunlimab alone or in combination with pembrolizumab in Japanese patients with advanced or metastatic solid tumors

**DOI:** 10.1007/s10147-026-03103-1

**Published:** 2026-06-30

**Authors:** Noboru Yamamoto, Gaurav Bajaj, Homer C. Adams, Ami Takahashi, Risa Fukushi, Ulf Forssmann, Edward Ramirez Ganoza, Yasutoshi Kuboki

**Affiliations:** 1 National Cancer Center Hospital, 5-1-1 Tsukiji, Chuo-Ku, Tokyo, 104-0045 Japan; 2 Genmab, Princeton, NJ USA; 3 Genmab K.K., Tokyo, Japan; 4 Genmab, Copenhagen, Denmark; 5 National Cancer Center Hospital East, Kashiwa, Japan

**Keywords:** Acasunlimab, Pembrolizumab, Solid malignancies, Pharmacokinetics, Safety, Japanese patients

## Abstract

**Background:**

Acasunlimab is a PD-L1 × 4-1BB bispecific antibody that induces T-cell activation through conditional 4-1BB stimulation, which is dependent on simultaneous PD-L1 binding, while the acasunlimab PD-L1–specific arm inhibits PD-1/PD-L1 activity without 4-1BB binding. Acasunlimab may produce prolonged, synergistic antitumor responses in patients with advanced solid malignancies.

**Methods:**

This phase 1, open-label, dose-escalation trial evaluated acasunlimab monotherapy and combination therapy with pembrolizumab in Japanese adult patients with advanced or metastatic solid tumors. Using a 3 + 3 design, patients received single intravenous acasunlimab infusions (15–800 mg every 3 weeks [Q3W]), or acasunlimab 100 mg plus pembrolizumab 200 mg Q3W. Primary endpoints included adverse events, dose-limiting toxicity (DLT), and pharmacokinetics. Pharmacodynamics and antitumor activity were also evaluated.

**Results:**

Eighteen patients were enrolled with diverse tumor types. Grade 3 treatment-related adverse events (TRAEs) occurred in 5 of 15 patients receiving acasunlimab monotherapy and 1 of 3 patients who received acasunlimab plus pembrolizumab. No grade 4 or 5 TRAEs occurred, and no DLTs or TRAEs leading to death at any monotherapy or combination dose were reported. Pharmacokinetics for acasunlimab in Japanese patients were comparable to those in non-Japanese patients from other clinical studies. Six patients receiving acasunlimab monotherapy experienced a best overall response of stable disease, as did one patient receiving acasunlimab plus pembrolizumab.

**Conclusions:**

Acasunlimab demonstrated a manageable safety profile, with comparable pharmacokinetics in Japanese and non-Japanese patients. Antitumor activity was demonstrated in a small number of patients with diverse tumor types.

**Supplementary Information:**

The online version contains supplementary material available at 10.1007/s10147-026-03103-1.

## Introduction

Cancer can evade immune-mediated destruction by suppressing the immune response within the tumor microenvironment, thereby promoting tumor progression [1, 2]. Disrupting this cycle by modulating immune checkpoints can reinvigorate the antitumor activity of immune cells and has been a focus for cancer therapeutics [2–5]. For example, the programmed death 1 protein (PD-1) receptor and its ligands, PD-L1 and PD-L2, support tumor growth by maintaining immune tolerance of T cells, preventing active immune responses against cancer cells [4, 6, 7]. Targeting the PD-(L)1 pathway became the new cancer treatment paradigm when blocking the negative regulatory signals of the pathway was shown to hinder immunosuppressive signals exploited by tumors for survival and growth [2, 8]. Checkpoint inhibitors (CPIs) that disrupt PD-1 signaling are now used to treat a broad range of solid malignancies, representing an important advancement in cancer management [9–14]. However, not all patients respond to CPIs; some patients respond transiently and progression following CPI therapy is common [2, 3]. Additional treatments are needed as treatment options become limited after progression on CPIs [15].

4-1BB is an inducible costimulatory receptor expressed on the surface of activated CD4 + and CD8 + T cells and activated natural killer cells that has been explored as a target for immunoregulatory therapies [2, 5]. When stimulated by its ligand on antigen-presenting cells, 4-1BB promotes T-cell survival and induces T-cell proliferation, cytokine production, and cytotoxic activity [2, 5, 16, 17]. In early-phase clinical studies, monoclonal antibodies (mAbs) that stimulated 4-1BB were associated with antitumor activity [5, 16, 18]. However, clinical development was hampered by a limited therapeutic window, with dose-limiting hepatoxicity occurring as, for example, with the strongly agonistic 4-1BB–targeting mAb urelumab [5, 16]. Development of next-generation 4-1BB agonists with targeted activity to tumor cells and improved tolerability is being actively pursued [5, 18].

Acasunlimab is an investigational PD-L1 × 4-1BB bispecific antibody inducing conditional activation of 4-1BB strictly dependent on simultaneous PD-L1 binding [5, 19]. Because 4-1BB is frequently co-expressed with PD-1 on CD8 + T cells, PD-(L)1 pathway blockade with co-stimulation of 4-1BB may produce synergistic effects on T-cell effector functions, extending the duration of the antitumor response [20, 21]. Preclinical and pharmacokinetic (PK)/pharmacodynamic (PD) findings support combining acasunlimab with pembrolizumab for optimal 4-1BB activation and more complete blockade of the PD-1/PD-(L)1 axis [5, 21]. Acasunlimab monotherapy data from the first-in-human phase 1 trial (NCT03917381) showed promising efficacy and a manageable safety profile in heavily pre-treated patients with solid tumors [5]. Disease control occurred in 40 of 61 patients at the time of data cutoff (65.6%; 95% CI, 52.3–77.3), with a median (range) follow-up of 9.4 (0.3–20.0) months [5]. Here, we describe the results of an open-label, phase 1 dose-escalation trial evaluating the safety of acasunlimab monotherapy or in combination with pembrolizumab in Japanese patients with advanced or metastatic non–central nervous system (non-CNS) solid tumors. The aim of the study was to determine whether the global dose and immune-mediated safety profile of acasunlimab can be extrapolated to Japanese patients.

## Patients and methods

### Study design and patient selection

This was an open-label, single-country, multicenter, phase 1 dose-escalation study evaluating the safety, tolerability, and PK of acasunlimab monotherapy or in combination with pembrolizumab in Japanese patients with advanced or metastatic non-CNS solid tumors, conducted between June 2021 and October 2024 (GCT1046-02; NCT04937153; JRCT2031210112). Patients were aged at least 20 years, of Japanese ethnicity, with histologically confirmed metastatic or unresectable non-CNS solid tumors, for whom there was no available standard therapy likely to confer clinical benefit. Additional inclusion criteria comprised (1) measurable disease per Response Evaluation Criteria in Solid Tumors (RECIST) version 1.1, (2) Eastern Cooperative Oncology Group performance status 0 to 1, (3) adequate renal, liver, and hematologic function, and (iv) adequate coagulation status. Patients were excluded if they had uncontrolled intercurrent illness (full eligibility criteria are in the Online Resource 1: Supplementary Methods).

### Study treatment

**Fig. 1:**
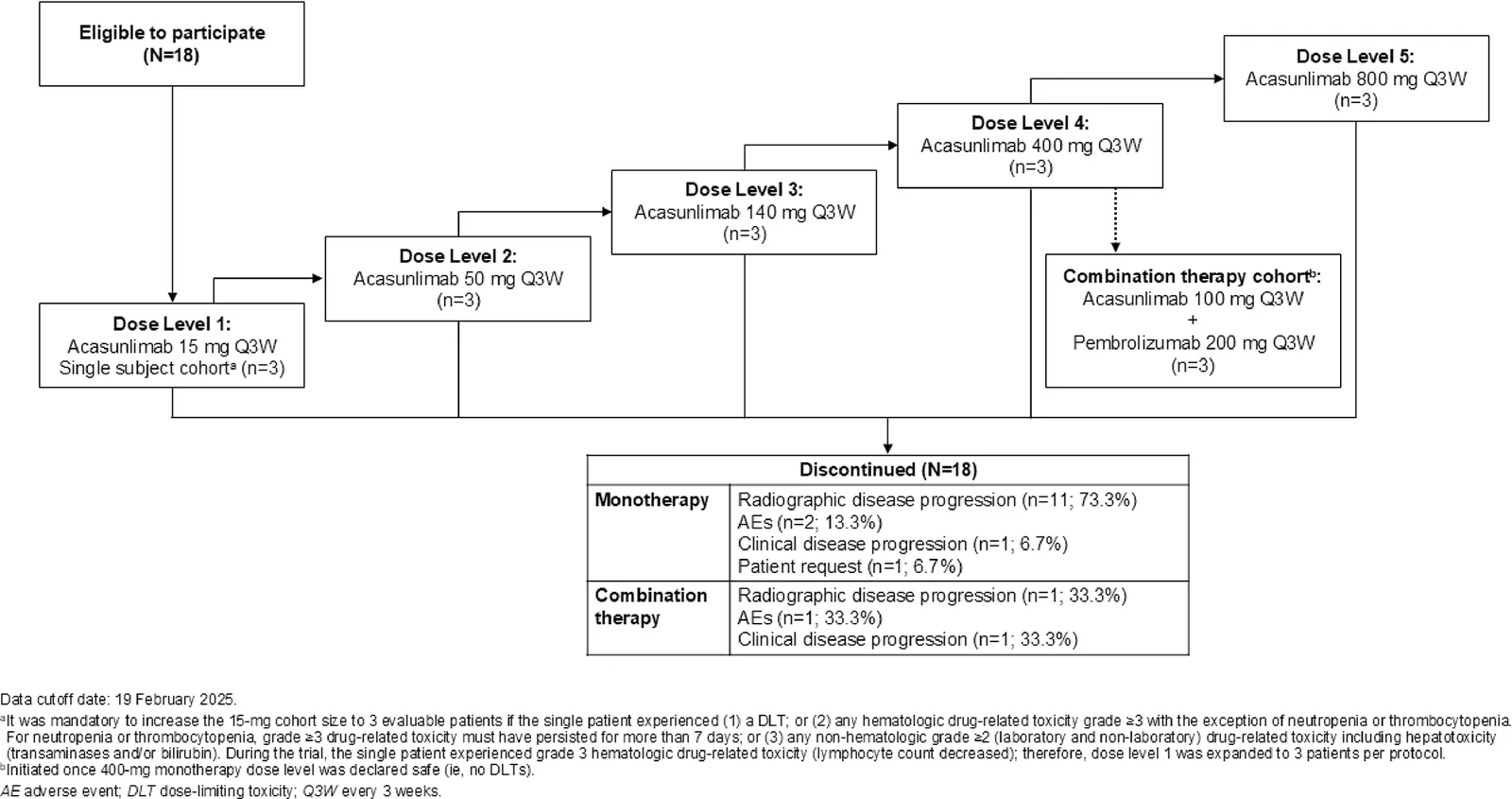
Study design. Eighteen patients were enrolled in the dose-escalation study using a 3 + 3 design. The acasunlimab plus pembrolizumab cohort was initiated once the 140-mg and 400-mg acasunlimab monotherapy doses were declared safe

Patients were sequentially assigned to treatment groups as openings became available. Patients assigned to acasunlimab monotherapy received single intravenous infusions of 15 mg, 50 mg, 140 mg, 400 mg, or 800 mg acasunlimab every 3 weeks (Q3W). Based on results from the first-in-human GCT1046-01 trial (doses ranging from 25 to 1200 mg Q3W were evaluated in 61 patients and were considered safe and generally well tolerated [5]) and recommendation from the Pharmaceuticals and Medical Devices Agency (Japan), the starting dose for this trial was determined to be 15 mg Q3W in the dose-escalation phase. Dose escalation began with an accelerated phase consisting of a single patient dosed at 15 mg (dose level 1) that was expanded to a 3-patient cohort per protocol. Dose levels 2, 3, 4, and 5 were administered sequentially using a 3 + 3 dose-escalation design. The acasunlimab 100 mg plus pembrolizumab 200 mg (Q3W) cohort was initiated once the 400-mg monotherapy dose was declared safe (Fig. 1). Patients assigned to the acasunlimab plus pembrolizumab group received pembrolizumab 200 mg first, followed by acasunlimab 100 mg. Acasunlimab and pembrolizumab were infused over at least 30 min and acasunlimab infusion began at least 30 min after the end of the pembrolizumab infusion. All patients continued treatment until disease progression, unacceptable toxicity, or another discontinuation criterion was met. Dose interruptions of acasunlimab were permitted according to prespecified guidelines.

### Outcome measures

The primary study endpoints were adverse events (AEs), dose-limiting toxicity (DLT), and PK parameters. Exploratory endpoints included evaluation of immune biomarkers to assess PD activity and antitumor activity of acasunlimab monotherapy and in combination with pembrolizumab. AEs were coded to the Medical Dictionary for Regulatory Activities version 27.1 and graded per National Cancer Institute Common Terminology Criteria for Adverse Events (NCI-CTCAE) version 5.0, with the exception of cytokine release syndrome, which was graded according to the American Society for Transplantation and Cellular Therapy criteria and relationship to trial drug. DLTs were evaluated in the first cycle of treatment (first 21 days) and graded according to NCI-CTCAE version 5.0. Venous blood samples for measurement of plasma concentrations of acasunlimab were collected before infusion, at the end of infusion, and 2 and 4 h after infusion on day 1; before infusion on days 2, 3, 8, and 15 of cycles 1 and 3; before infusion and at the end of infusion on day 1 of cycles 2, 5, and all subsequent cycles; and at treatment discontinuation.

PK data from cycles 1 and 3 were used to estimate model parameters associated with describing the PK of acasunlimab. PK parameters were calculated based on non-compartmental analyses. A population PK–based approach was also used to compare individual PK parameters including the maximum drug concentration (C_max_) after a single dose, and area under the concentration–time curve (AUC) in Japanese and non-Japanese patients. A preliminary population PK model was developed to characterize acasunlimab PK using data from this study, GCT1046-02, and other global trials including GCT1046-01 and GCT1046-04. The lower limit of quantification (LLOQ) was 0.1 µg/mL.

Exploratory PD analyses were performed using peripheral blood, including measurements of key cytokines and chemokine levels. Blood samples were collected at baseline, during cycle 1, and at each cycle thereafter (days 1, 2, 3, and 8 in cycle 1; days 1 and 8 in cycles 2 and 3; and day 1 in cycle 4 and beyond). Plasma samples were analyzed using validated methods including an enzyme-linked immunosorbent assay method with electrochemiluminescence detection.

Antitumor activity was investigator-assessed based on RECIST version 1.1 and included (1) confirmed objective response rate, defined as the proportion of patients with a best overall response of complete response (CR) or partial response (PR), which was confirmed by subsequent tumor assessment; (2) disease control rate; and (3) duration of response (DOR). DOR was calculated as the date of the first documented response of CR or PR until the date of first documented disease progression or death. Antitumor activity was assessed by imaging every 9 weeks (± 7 days) until disease progression was determined by the investigator; the start of new anticancer therapy; withdrawal of consent; or death.

### Statistical analysis

The sample size for the monotherapy group was driven by the 3 + 3 design and prespecified dose levels. For the combination therapy cohort, a decision table derived from a Bayesian optimal interval design with a target toxicity rate of 0.3 determined the sample size. For the combination cohort, at least 3 and no more than 4 DLT-evaluable patients were to be assessed initially, and up to 6 additional patients could be enrolled. Patients not evaluable for DLTs could be replaced at any time. As a result, it was planned to treat between 16 and 39 patients. All safety and efficacy analyses were performed on the full analysis set, which included all patients receiving at least 1 dose of acasunlimab, and the exact 95% CIs were calculated by the Clopper-Pearson method. The PK analysis set included all patients with an evaluable PK profile (who received ≥ 1 dose of treatment and for whom ≥ 1 PK parameter was available).

AEs and laboratory values were summarized descriptively. Biomarker assessments were performed in patients who had samples collected and with which a test was performed, with results summarized by time point using descriptive statistics.

## Results

### Baseline characteristics and treatment exposure

At the data cutoff (19 February 2025), 18 patients who met eligibility criteria were enrolled. Most patients were male, the median age was 61.0 years, and 60% had received at least 3 prior lines of therapy (Table 1). Tumor types included bladder cancer; chondrosarcoma; colorectal cancer; gallbladder cancer; leiomyosarcoma; non–small cell lung cancer (NSCLC); ovarian cancer; parotid cancer; rectal cancer; renal cancer; thymus cancer (n = 1 each); cervical cancer (n = 2); and pancreatic cancer (n = 5). Median exposure was 2.1 months (interquartile range [IQR], 2.1–4.1) among patients in the monotherapy groups (n = 15) and 1.5 months (IQR, 0.7–2.1) among patients receiving acasunlimab plus pembrolizumab (n = 3). All 18 patients discontinued study treatment by the data cutoff due to radiographic disease progression (n = 11; 73.3%), AEs (n = 2; 13.3%), clinical disease progression (n = 1; 6.7%), and patient request (n = 1; 6.7%) with acasunlimab monotherapy, and radiographic disease progression, clinical progression, and AEs (all n = 1; 33.3%) with acasunlimab plus pembrolizumab (Fig. 1).

**Table 1:** Baseline characteristics and treatment exposures

	Acasunlimab Q3W monotherapy^a^ (n = 15)	Acasunlimab 100 mg Q3W + pembrolizumab 200 mg Q3W (n = 3)
Age		
Median age (min, max), years	61.0 (35, 75)	61.0 (39, 76)
< 65 years, n (%)	9 (60.0)	2 (66.7)
≥ 65 years, n (%)	6 (40.0)	1 (33.3)
Sex, n (%)		
Male	8 (53.3)	3 (100.0)
Female	7 (46.7)	0
BMI		
Median BMI (min, max), kg/m^2^	21.9 (16.8, 40.5)	24.6 (20.3, 26.1)
Disease stage at entry, n (%)		
IV	13 (86.7)	2 (66.7)
IVA	0	1 (33.3)
IVB	2 (13.3)	0
Tumor type, n (%)		
Bladder	1 (6.7)	0
Cervical	2 (13.3)	0
Colorectal	1 (6.7)	0
Non–small cell lung	0	1 (33.3)
Ovarian	1 (6.7)	0
Pancreatic	4 (26.7)	1 (33.3)
Renal	1 (6.7)	0
Other^b^	5 (33.3)	1 (33.3)
ECOG PS, n (%)		
0	12 (80.0)	1 (33.3)
1	3 (20.0)	2 (66.7)
Prior systemic anticancer regimens, n (%)		
1	3 (20.0)	0
2	2 (13.3)	1 (33.3)
3	1 (6.7)	1 (33.3)
> 3	9 (60.0)	1 (33.3)
Prior CPI (any combination), n (%)		
Pembrolizumab	2 (13.3)	0
Nivolumab	2 (13.3)	0
Durvalumab	0	0
Atezolizumab	1 (6.7)	1 (33.3)
Missing or not available	10 (66.7)	2 (66.7)

Data cut-off date: 19 February 2025

^a^Single intravenous infusions of 15, 50, 140, 400, or 800 mg

^b^Other tumor types included: leiomyosarcoma (n = 1), gallbladder cancer (n = 1), parotid cancer (n = 1), thymus cancer (n = 1), and rectal cancer (n = 1) for acasunlimab monotherapy; chondrosarcoma (n = 1) for acasunlimab plus pembrolizumab

*BMI* body mass index; *CPI* checkpoint inhibitor; *ECOG PS* Eastern Cooperative Oncology Group performance status; *max* maximum; *min* minimum; *Q3W* every 3 weeks

### Safety

Sixteen patients experienced treatment-emergent AEs (acasunlimab monotherapy: n/N = 13/15 [86.7%]; acasunlimab plus pembrolizumab: n/N = 3/3 [100%]) and 13 experienced treatment-related AEs (TRAEs) (acasunlimab monotherapy: n/N = 12/15 [80%]; acasunlimab plus pembrolizumab: n/N = 1/3 [33.3%]) (Table 2). The most common TRAEs were pyrexia (33.3%), hepatic events (26.7%), pruritus (20.0%), decreased platelet counts (20.0%), and decreased white blood cell (WBC) counts (20.0%). Five patients (33.3%) receiving acasunlimab monotherapy experienced grade 3 TRAEs; these included 3 events of immune-mediated hepatitis in the 140-mg group, 2 events of decreased lymphocyte count (1 each in the 15-mg and 140-mg groups), 1 event of neutrophil cell count decrease in the 15-mg group, and 1 event of decreased WBC count in the 15-mg group. These TRAEs were managed by dose delay. No grade 4 or 5 TRAEs were reported, and there were no dose-dependent increases in the incidence or severity grade of TRAEs. There were no DLTs or TRAEs leading to death with monotherapy or combination treatment.

**Table 2:** Overview of adverse events

	Acasunlimab Q3W monotherapy^a^ (n = 15)	Acasunlimab 100 mg Q3W + pembrolizumab 200 mg Q3W (n = 3)
Patients with ≥ 1 TEAE, n (%)	13 (86.7)	3 (100.0)
Grade 3	7 (46.7)	2 (66.7)
Grade 4	0	0
Grade 5	0	0
Patients with serious TEAEs, n (%)	2 (13.3)	2 (66.7)
TEAE leading to treatment withdrawal	2 (13.3)	1 (33.3)
Patients with TRAEs, n (%)	12 (80.0)	1 (33.3)
Grade 3^b^	5 (33.3)	1 (33.3)
Grade 4	0	0
Grade 5	0	0
Patients with serious TRAEs, n (%)	2 (13.3)	1 (33.3)
DLT in cycle 1	0	0
Most common TRAEs (≥ 10%) by preferred term, n (%)		
Pyrexia	5 (33.3)	0
Hepatic events^c^	4 (26.7)	0
Pruritus	3 (20.0)	0
Decreased platelet count	3 (20.0)	0
Decreased WBC count	3 (20.0)	0
Decreased lymphocyte count	2 (13.3)	0
Rash	2 (13.3)	0
Rash maculopapular	2 (13.3)	0
Stomatitis	2 (13.3)	0
Hemophagocytic lymphohistiocytosis	0	1 (33.3)

Data cut-off date: 19 February 2025

^a^Single intravenous infusions of 15, 50, 140, 400, or 800 mg

^b^Grade 3 TRAEs included immune-mediated hepatitis in 3 patients (20.0%), lymphocyte count decrease in 2 patients (13.3%), and neutrophil cell count decrease and WBC count decrease in 1 patient each (6.7%). One 76-year old male patient with stage IV NSCLC and other relevant medical history including hypertension, type 2 diabetes mellitus, and hypothyroidism treated with acasunlimab plus pembrolizumab experienced grade 3 HLH. Prior anticancer therapies received included atezolizumab plus carboplatin and paclitaxel, and docetaxel. The subject received acasunlimab 100 mg IV preceded by pembrolizumab 200 mg IV per protocol on day 1 and day 25 (last dose of trial drug). On day 35, the subject was hospitalized due to grade 3 HLH and was treated with prednisolone, methylprednisolone, and IV fluids (calcium chloride dihydrate, glucose, potassium chloride, sodium chloride, sodium lactate). The event improved to grade 1 by day 60 and the subject was discharged from the hospital on day 61. At the end of trial visit on day 109, the event of HLH was considered recovered/resolved

^c^Hepatic events included immune-related hepatitis and increased alanine aminotransferase, aspartate aminotransferase, and gamma-glutamyl transferase

*DLT* dose-limiting toxicity; *HLH* hemophagocytic lymphohistiocytosis; *IV* intravenous; *NSCLC*, non–small cell lung cancer; *TEAE* treatment-emergent adverse event; *TRAE* treatment-related adverse event; *Q3W* every 3 weeks; *WBC* white blood cell

Two patients treated with acasunlimab 140 mg monotherapy discontinued treatment due to TRAEs; both discontinuations were for grade 3 immune-mediated hepatitis considered related to acasunlimab. One patient treated with acasunlimab plus pembrolizumab experienced grade 3 hemophagocytic lymphohistiocytosis (HLH), which was deemed related to acasunlimab and pembrolizumab treatment. Further administration of the investigational treatment was discontinued, and the HLH resolved with corticosteroid treatment.

### Pharmacokinetics

**Fig. 2:**
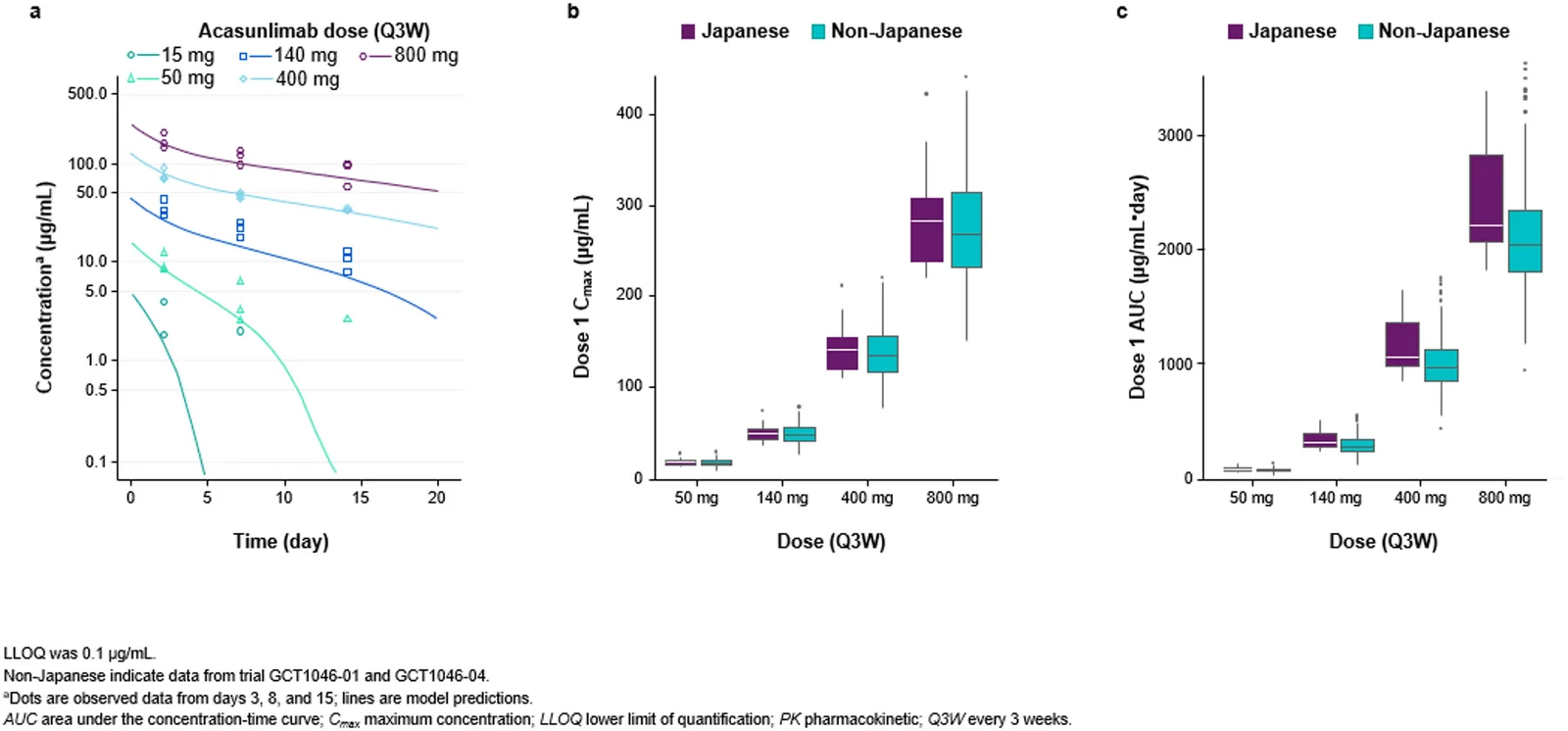
Observed and model-predicted acasunlimab PK profile in Japanese patients after first dose of acasunlimab monotherapy given every 3 weeks (n = 14) at doses evaluated in this trial (GCT1046-02) **a**. Comparison of model-predicted exposures with acasunlimab monotherapy in Japanese and non-Japanese patients: C_max_ **b** and AUC **c** after first dose

The PK analysis set included 14 patients who received acasunlimab monotherapy. Acasunlimab C_max_ values were observed shortly after the end of infusion in the PK analysis set (Fig. 2a). The acasunlimab PK profile was determined using non-compartment analyses with linear and nonlinear clearance, and the model described the data well. Nonlinear clearance was driven by target-mediated elimination, described by Michaelis–Menten kinetics. C_max_, trough plasma concentration (C_trough_), area under the concentration–time curve from time zero to last measurable concentration (AUC_last_), and half-life (t_1/2_) values for cycle 1 and cycle 3 are included in Online Resource: Table S1. There was minimal or no accumulation observed with repeat acasunlimab dosing at less than 200 mg Q3W. Considering acasunlimab is also eliminated via target-mediated drug disposition, terminal t_1/2_ determined using non-compartmental analyses is not constant and increased with dose, and does not accurately characterize disappearance of the drug from systemic circulation.

Comparison of model-predicted PK parameters in Japanese and non-Japanese patients showed a clear overlap in C_max_ after the first dose with an approximately 6% difference between Japanese and non-Japanese patients (Fig. 2b and Online Resource: Table S2). Model-predicted AUCs were relatively higher in Japanese patients compared with evaluation in non-Japanese study patients, but the difference was less than 20% (Fig. 2c and Online Resource: Table S2).

### PD activity and antitumor activity

**Fig. 3:**
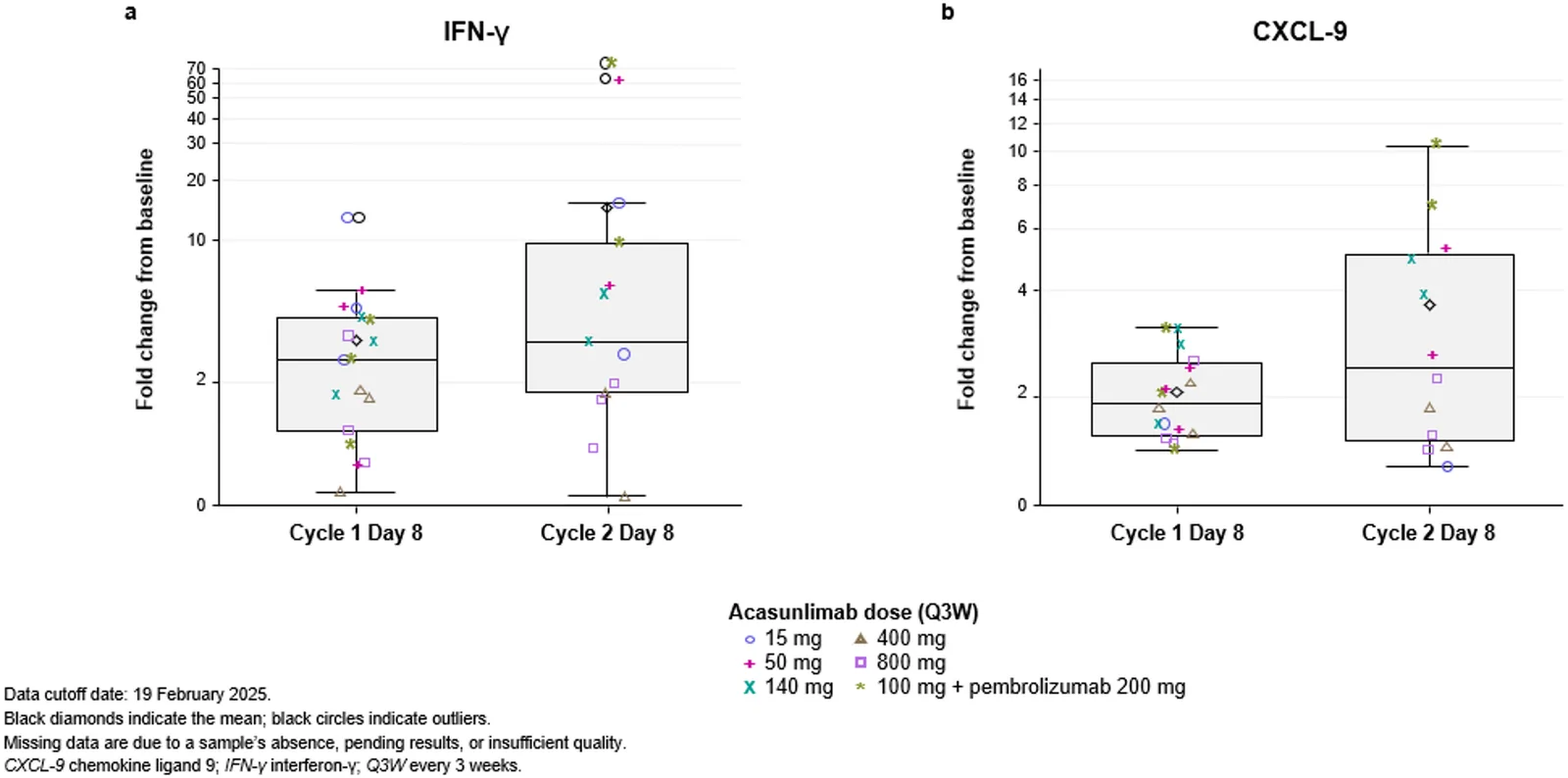
Pharmacodynamic activity of different doses of acasunlimab and acasunlimab in combination with pembrolizumab. Fold change from baseline in IFN-γ in peripheral blood measured at cycle 1 day 8 and cycle 2 day 8 **a**. Fold change from baseline in CXCL-9 in peripheral blood measured at cycle 1 day 8 and cycle 2 day 8 **b**

**Fig. 4:**
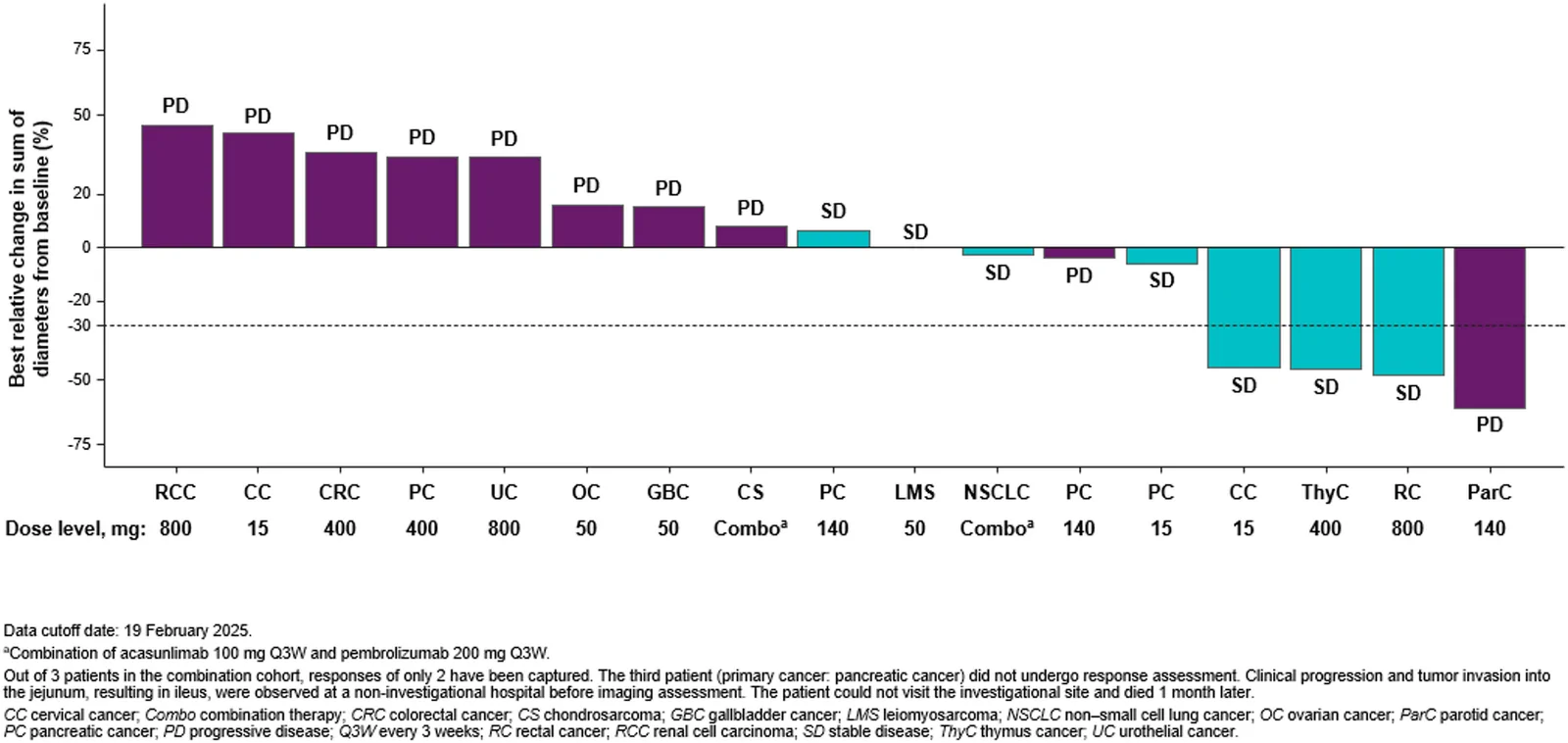
Antitumor activity of acasunlimab. Best change from baseline in tumor responses following treatment with acasunlimab monotherapy and acasunlimab plus pembrolizumab

Consistent with the proposed mechanism of acasunlimab, increased levels of the peripheral blood PD markers interferon-γ (IFN-γ) and chemokine ligand 9 (CXCL-9) were observed post-treatment (Fig. 3). Lower dose levels (≤ 140 mg) were generally associated with greater increases in peripheral immune modulation than higher dose levels (≥ 400 mg) in the first 2 cycles. Antitumor responses were available from 17 patients in the full analysis set (Fig. 4). One patient with pancreatic cancer treated with acasunlimab plus pembrolizumab experienced clinical progression and tumor invasion into the jejunum, detected before an imaging assessment could occur, and the patient passed away 1 month later. Although 3 patients achieved a PR with acasunlimab monotherapy at 15-mg, 400-mg, and 800-mg dose levels, they experienced subsequent progression (with or without new lesions) or progression of nontarget lesions. Thus, no confirmed responses were observed. The disease control rate was 40% (n/N = 6/15; 95% CI, 16.3%-67.7%) for patients receiving acasunlimab monotherapy and 33% (n/N = 1/3; 95% CI, 0.8%-90.6%) for patients receiving acasunlimab plus pembrolizumab. Six patients receiving acasunlimab monotherapy (15 mg, n = 2; 50 mg, 140 mg, 400 mg, and 800 mg, n = 1 each) and 1 patient receiving acasunlimab plus pembrolizumab 200 mg experienced a best overall response of SD. One patient with leiomyosarcoma treated with acasunlimab 50 mg Q3W maintained SD for more than 33 months.

## Discussion

In this phase 1 dose-escalation trial in Japanese patients with advanced solid tumors, acasunlimab monotherapy or in combination with pembrolizumab demonstrated manageable safety and displayed PK comparable with PK observed in other clinical studies of acasunlimab in non-Japanese patients. No patients treated with acasunlimab monotherapy up to 800 mg Q3W or acasunlimab plus pembrolizumab experienced AEs considered to be DLTs, and the maximum tolerated dose was not reached, supporting the tolerability of acasunlimab in Japanese patients.

Overall, the safety profile reported here is consistent with the first-in-human trial of acasunlimab. Five patients who received monotherapy and 1 patient who received combination therapy experienced grade 3 TRAEs. In the monotherapy group, these grade 3 TRAEs included hepatic events and decreased hematologic values and were managed by dose delay. The patient who experienced HLH in the combination therapy group discontinued study treatment and was immediately treated with corticosteroids, which resulted in full recovery. HLH is a serious but relatively rare immune-related AE that can occur with therapies targeting immune effector cells, including chimeric antigen receptor T-cell therapies, bispecific antibodies, and CPIs, and prompt recognition and intervention are recommended to improve outcomes for patients who develop HLH secondary to immunotherapy [22–24].

The safety of acasunlimab compares favorably with that of urelumab, the first-generation 4-1BB agonist that had development discontinued due to 2 deaths resulting from treatment-related hepatoxicity at doses associated with antitumor activity (≥ 1 mg/kg) [25, 26]. Muik et al. noted that the comparatively favorable safety profile of acasunlimab may be due to its 4-1BB agonist activity being conditional on PD-L1 binding, which may limit widespread T-cell–mediated cytotoxicity [5]. Overall, while the liver toxicity events appear to be more manageable compared with events with earlier-generation 4-1BB agonists, liver toxicity associated with acasunlimab monotherapy and combination therapy will be important to monitor in larger trials [26].

Model-predicted acasunlimab concentrations from the preliminary model used to characterize PK parameters aligned with observed PK data. Comparison of model-predicted PK parameters in Japanese and non-Japanese patients showed an overlap for C_max_ with a 6% difference between Japanese and non-Japanese patients. The data also show an overlap of AUC after the first dose; however, AUC was relatively higher in Japanese patients (< 20%) than in non-Japanese patients. This difference in PK parameters is likely due to the lower average body weight of Japanese patients compared with the average weight in the global population, an observation often noted in phase 1 studies of mAbs in Japanese patients (eg, the body weight difference between Japanese patients and Caucasian patients is approximately 20%) [27]. As the difference in PK parameters was < 20%, the PK parameters in Japanese patients after acasunlimab monotherapy were not considered to be substantially different from those in non-Japanese patients.

Early modulation of peripheral cytokines, as reflected by increased secretion of peripheral IFN-γ and IFN-γ–inducible chemokine CXCL-9 following treatment with acasunlimab, is characteristic of T-cell activation and is consistent with the acasunlimab mechanism of action that supports its biologic activity. Acasunlimab demonstrated promising antitumor activity, and although these outcomes were SD, these are encouraging results for this small population with heavily pretreated and/or difficult-to-treat cancers.

Limitations of the current study, consistent with other phase 1 studies, include the small number of patients, particularly in the acasunlimab plus pembrolizumab group; the open-label and single-arm study design; and the short duration of the study. The small sample size also limits determinations regarding the causality of observed hepatic events and HLH.

Importantly, once patients’ disease progresses on or following CPI therapy, treatment options are limited, and patients face significant drawbacks with the remaining options, including disappointing efficacy and significant toxicity. There is therefore an unmet need for effective treatments for these patients with advanced cancer [5].

The study results are consistent with the phase 1/2a GCT1046-01 (NCT03917381) and phase 2 GCT1046-04 (NCT05117242) studies, demonstrating a manageable safety profile for acasunlimab, with PK comparable between Japanese and non-Japanese patients. Consistent with previous studies, increases in peripheral cytokines after acasunlimab administration suggest T-cell activation, supporting the proposed mechanism of action of acasunlimab in stimulating the antitumor response.

## Supplementary Information

Below is the link to the electronic supplementary material.

Supplementary Material 1

## Data Availability

The data included in this study are not publicly available but may be available upon reasonable request from the corresponding author with institutional approval.

## Declarations

### Previous presentation

Data from this study were previously presented at the Japanese Society of Medical Oncology Annual Meeting, 6–8 March 2025, Kobe, Japan.
