# Supplementary material for: A phase 1 dose-finding and pharmacokinetic study of acasunlimab alone or in combination with pembrolizumab in Japanese patients with advanced or metastatic solid tumors
Source: Int J Clin Oncol. 2026 Jun 30;31(9):1928–36. doi: 10.1007/s10147-026-03103-1 (PMC13506613; doi:10.1007/s10147-026-03103-1)
Supplement: Supplementary file 1 [file 10147_2026_3103_MOESM1_ESM.docx]

**Online Resource Material for:**

**A phase 1 dose-finding and pharmacokinetic study of acasunlimab alone or in combination with pembrolizumab in Japanese patients with advanced or metastatic solid tumors**

***International Journal of Clinical Oncology***

Noboru Yamamoto, MD, PhD,^1^ Gaurav Bajaj, PhD,^2^ Homer C. Adams III, PhD,^2^

Ami Takahashi, PhD,^3^ Risa Fukushi, PhD,^3^ Ulf Forssmann, MD,^4^ Edward Ramirez Ganoza, MD,^3^ Yasutoshi Kuboki, MD, PhD^5^

^1^National Cancer Center Hospital, Tokyo, Japan

^2^Genmab, Plainsboro, NJ, USA

^3^Genmab K.K., Tokyo, Japan

^4^Genmab, Copenhagen, Denmark

^5^National Cancer Center Hospital East, Kashiwa, Japan

**Corresponding author:** Noboru Yamamoto, MD, PhD; email: nbryamam@ncc.go.jp

**Online Resource 1: Supplementary Methods**

**Online Resource 2: Table S1**

**Online Resource 2: Table S2**

**Online Resource 1: Supplementary Methods**

| **Full Eligibility Criteria** | |
| --- | --- |
| Inclusion criteria | Each subject must fulfill all the following criteria to be treated in the trial.   1. Subject must sign an informed consent form (ICF) indicating that he or she understands the purpose of and procedures required for the trial and is willing to participate in the trial prior to any trial-related assessments or procedures. 2. Subject must have a histologically confirmed non–central nervous system (CNS) solid tumor that is metastatic or unresectable and for whom there is no available standard therapy likely to confer clinical benefit; or a subject who is not a candidate for such available therapy and for whom, in the opinion of the investigator, experimental therapy with acasunlimab or acasunlimab in combination with pembrolizumab may be beneficial. 3. Subject must be ≥ 20 years of age. 4. Asian race and Japanese ethnicity. 5. Subject must have measurable disease according to Response Evaluation Criteria in Solid Tumors (RECIST) 1.1 6. Subject must have Eastern Cooperative Oncology Group (ECOG) performance status (PS) ≤ 1. 7. Subject must have organ and bone marrow function as follows:    a. Bone marrow/hematological function:       i. Absolute neutrophil count (ANC) ≥ 1.5 × 10^9^/L       ii. Hemoglobin ≥ 9.0 g/dL       iii. Platelet count ≥ 100 × 10^9^/L    b. Liver function:       i. Total bilirubin ≤ upper limit of normal (ULN)       ii. Alanine aminotransferase (ALT) ≤ 1.5 × ULN       iii. Aspartate aminotransferase (AST) ≤ 1.5 × ULN       iv. Albumin ≥ 30 g/L    c. Coagulation status:       i. Prothrombin time (PT)/international normalized ratio ≤ 1.5       ii. Activated partial thromboplastin time (aPTT) ≤ 1.5 × ULN Note: Subjects receiving anticoagulant therapies should have the PT and aPTT within the therapeutic range of intended use of anticoagulants.    d. Renal function: Glomerular filtration rate (GFR) ≥ 45 mL/min/1.73 m², according to the abbreviated Modification of Diet in Renal Disease equation: GFR = 186 × (SCr - 1.154) × (age - 0.203), where SCr, the serum creatinine level, is expressed in mg/dL; multiply the result by 0.742 if the subject is female. 8. A woman of childbearing potential (WOCBP) (postmenopausal or permanently sterilized is considered as not having reproductive potential) must agree to use adequate contraception during the trial and for 6 months after the last trial treatment administration. Adequate contraception is defined as highly effective methods of contraception. 9. A WOCBP must have a negative serum pregnancy test (beta-human chorionic gonadotropin [β-hCG]) or urine pregnancy test at screening. 10. A woman must agree not to donate eggs (ova, oocytes) for the purposes of assisted reproduction during the trial and for 6 months after the last dose of trial treatment. 11. A man who is sexually active with a WOCBP and has not had a vasectomy must agree to use a barrier method of birth control, i.e., condom with a partner using an adequate contraception and must also not donate sperm during the trial and for 6 months after receiving the last dose of trial treatment. 12. Subject must provide a tumor tissue sample (formalin-fixed paraffin-embedded [FFPE] blocks/slides) from archival tissue or fresh biopsy collected before C1D1, preferably derived from advanced disease stage. |
| Exclusion criteria | Any potential subject who meets any of the following criteria will be excluded from being treated in the trial.   1. Subject has uncontrolled intercurrent illness, including but not limited to:    a. Ongoing or active infection requiring IV treatment with anti-infective therapy that has been administered less than 2 weeks prior to first dose or any ongoing systemic inflammatory condition requiring further diagnostic work-up or management during screening.    b. Symptomatic congestive heart failure (Grade III or IV as classified by the New York Heart Association), unstable angina pectoris or cardiac arrhythmia.    c. Uncontrolled hypertension defined as systolic blood pressure ≥ 160 mm Hg and/or diastolic blood pressure ≥ 100 mm Hg, despite optimal medical management.    d. Ongoing or recent (within 1 year) evidence of significant autoimmune disease that required treatment with systemic immunosuppressive treatments, which may suggest risk for immune-related adverse events (irAEs). ***Note: The following conditions are not exclusionary:*** Residual hypothyroidism that required only hormone replacement or psoriasis that does not require systemic treatment.    e. History of grade 3 or higher irAEs that led to treatment discontinuation of a checkpoint inhibitor (CPI) should be excluded. A subject with irAEs below grade 3 that led to discontinuation should be discussed with the sponsor.    f. Prior history of myositis, Guillain-Barré syndrome, or myasthenia gravis of any grade.    g. History of chronic liver disease or evidence of hepatic cirrhosis.    h. Evidence of interstitial lung disease (ILD).    i. History of non-infectious pneumonitis that has required steroids; or currently has pneumonitis.    j. History of organ allograft (except for corneal transplant) or autologous or allogeneic bone marrow transplant, or stem cell rescue within 3 months prior to the first dose of trial treatment.    k. Serious non-healing wound, skin ulcer (of any grade), or bone fracture. 2. Any history of intracerebral arteriovenous malformation, cerebral aneurysm, new (younger than 6 months) or progressive brain metastases or stroke.    a. Transient ischemic attack > 1 month prior to screening is allowed.    b. A subject with brain metastases must not be undergoing acute corticosteroid therapy or steroid taper. Chronic steroid therapy is acceptable provided that the dose is stable for the last 14 days prior to screening (≤ 10 mg prednisone daily or equivalent).    c. A subject with CNS symptoms should undergo a computed tomography (CT) scan or magnetic resonance imaging (MRI) of the brain to exclude new or progressive brain metastases. Spinal cord metastasis is acceptable. However, subjects with spinal cord compression should be excluded. 3. Prior therapy:    a. Radiotherapy: Radiotherapy within 14 days prior to the first dose of trial treatment. Palliative radiotherapy will be allowed.    b. Treatment with an anti-cancer agent (within 28 days or after at least 5 half-lives of the drug, whichever is shorter), prior to trial treatment administration. Accepted exceptions are bisphosphonates (e.g., pamidronate, zoledronic acid) and denosumab.    c. Has received any investigational agent (including investigational vaccines) or used an invasive investigational medical device within 28 days before the planned first dose of trial treatment or is currently enrolled in an interventional trial. (***Note:*** A subject who is in the follow-up phase of an interventional trial may participate if the subject has not received the investigational agent within 28 days of the first dose of the trial treatment.)    d. Prior treatment with live attenuated vaccines within 3 weeks prior to initiation of trial treatment. Chronic systemic immunosuppressive corticosteroid doses, i.e., prednisone > 10 mg daily (or equivalent) or a cumulative dose > 150 mg prednisone (or equivalent) within 14 days before the first trial treatment administration. Replacement therapy (e.g., thyroxine, insulin, or physiologic corticosteroid replacement therapy for adrenal or pituitary insufficiency) is not considered a form of systemic treatment and is permitted.    e. Has received granulocyte-colony stimulating factor (G-CSF) or granulocyte/macrophage colony stimulating factor (GM-CSF) support 4 weeks prior to the first trial treatment administration or is chronically transfusion dependent.    f. History of ≥ grade 3 allergic reactions to mAb therapy as well as known or suspected allergy or intolerance to any agent given during the course of this trial.    g. Discontinued treatment due to disease progression within the first 6 weeks of a CPI-containing treatment.    h. Prior treatment with a 4-1BB (CD137) targeted agent.    i. Prior treatment with a T-cell agonist or an anti-cytotoxic T-lymphocyte-associated protein-4 targeted agent within 12 weeks prior to the initiation of treatment. 4. Toxicities from previous anti-cancer therapies that have not resolved to baseline levels or to grade 1 or less with the exception of alopecia, anorexia, vitiligo, fatigue, hyperthyroidism, hypothyroidism, and peripheral neuropathy. Anorexia, hyperthyroidism, hypothyroidism, and peripheral neuropathy must have recovered to ≤ grade 2. 5. Known past or current malignancy other than inclusion diagnosis, except for:    a. Cervical carcinoma of Stage 1B or less.    b. Non-invasive basal cell or squamous cell skin carcinoma.    c. Non-invasive, superficial bladder cancer.    d. Prostate cancer with currently undetectable prostate-specific antigen.    e. Any curable cancer with a CR of > 2 years’ duration. 6. Known allergies, hypersensitivity, or intolerance to the trial treatment or its excipients. 7. Any condition for which, in the opinion of the investigator, participation would not be in the best interest of the subject (e.g., compromise the well-being) or that could prevent, limit, or confound the protocol-specified assessments. 8. Has had major surgery (e.g., requiring general anesthesia) within 4 weeks before screening, or will not have fully recovered from surgery, or has surgery planned during the time the subject is expected to participate in the trial. **Note:** A subject with a planned surgical procedure to be conducted under local anesthesia may participate. 9. Known history of seropositivity for human immunodeficiency virus (HIV). 10. Subject is known to be hepatitis B positive (defined by a positive test for hepatitis B surface antigen [HBsAg] or antibodies to hepatitis B surface or core antigens. Subjects immune due to vaccination or passive immunization due to immunoglobulin therapy can be included. 11. Known medical history or ongoing hepatitis C infection that has not been cured. 12. Substance abuse, medical, psychological, or social conditions that may interfere with the subject’s participation in the trial or evaluation of the trial result. 13. Subject has been dosed before in this trial. 14. Female subject is pregnant, or breast feeding and cannot discontinue breast feeding for the duration of the trial or at least 6 months after the last trial treatment administration or intends to conceive children within 6 months of ending trial treatment. 15. Subject in the combination cohort who has ongoing grade ≥ 2 sensory or motor neuropathy. 16. Subject in the combination cohort who has contraindications to the use of pembrolizumab per local prescribing information. |

**Online Resource 2: Table S1** Acasunlimab PK parameters, evaluated for cycles 1 and 3 at dose levels 15 mg to 800 mg given every 3 weeks, using non-compartmental analyses

| **Dose (mg)** | **C_max_ (μg/mL),**  **geometric mean**  **(geometric CV%)** | | **C_trough_ (μg/mL),**  **geometric mean**  **(geometric CV%)** | | **AUC_last_ (μg/mL•day),**  **geometric mean**  **(geometric CV%)** | | **t_½_ (days), mean**  **(SD)** | |
| --- | --- | --- | --- | --- | --- | --- | --- | --- |
|  | **Cycle 1** | **Cycle 3** | **Cycle 1** | **Cycle 3** | **Cycle 1** | **Cycle 3** | **Cycle 1** | **Cycle 3** |
| **15** | 6.3  (52.4)  (n = 2) | 5.5  (38.1)  (n = 2) | NE | NE | 12.6  (128.4)  (n = 2) | 8.0  (43.3)  (n = 2) | 3.3  (2.4)  (n = 2) | 2.5  (1.1)  (n = 2) |
| **50** | 19.4  (23.3)  (n = 3) | 16.8  (30.1)  (n = 3) | 0.1  (NE)  (n = 3) | NE | 66  (53.4)  (n = 3) | 49.8  (43.4)  (n = 3) | 3.2  (0.4)  (n = 3) | 3.8  (0.8)  (n = 3) |
| **140** | 55.8  (5.2)  (n = 3) | 55.6  (NE)  (n = 1) | 4.7  (NE)  (n = 1) | NE | 350.6  (21.9)  (n = 3) | 460.5  (NE)  (n = 1) | 6.9  (1.8)  (n = 3) | 9.0  (NE)  (n = 1) |
| **400** | 163.6  (11.8)  (n = 3) | 196.5  (10.1)  (n = 2) | 29.0  (21.6)  (n = 3) | NE | 1027.9  (7.1)  (n = 3) | 1278.3  (7)  (n = 2) | 13.1  (2.0)  (n = 2) | 9.4  (1)  (n = 2) |
| **800** | 303.5  (16.7)  (n = 3) | 407  (21.6)  (n = 3) | 64.4  (13.7)  (n = 3) | NE | 2382.2  (18.5)  (n = 3) | 2949.3  (27.2)  (n = 3) | 13.1  (3)  (n = 3) | 14.4  (6.2)  (n = 3) |
| **Acasun 100/**  **pembro 200** | 33.1  (26.5)  (n = 3) | 21.4  (NE)  (n = 1) | 0.1  (NE)  (n = 2) | NE | 114.8  (18.8)  (n = 3) | 69.5  (NE)  (n = 1) | 3.5  (1)  (n = 3) | 4.1  (NE)  (n = 1) |

Geometric CV% = coefficient of variation = sqrt (exp [variance for log-transformed data] - 1) × 100.

The lower limit of quantification was 0.1 ug/mL.

*Acasun* acasunlimab; *AUC_last_* area under the concentration-time curve from time zero to time of last measurable concentration; *C_max_* maximum drug concentration after a single dose; *C_trough_* trough plasma concentration; *CV* coefficient of variation; *NE* not evaluable; *pembro* pembrolizumab; *t_1/2_* half-life.

**Online Resource 3: Table S2** Comparison of model-predicted PK parameters of acasunlimab after the first dose in Japanese and non-Japanese patients using population PK model

| **Dose (mg)** | **PK parameter** | **Japanese^a^** | **Non-Japanese^a^** | **% Difference compared with non-Japanese** |
| --- | --- | --- | --- | --- |
| **50** | AUC (μg/mL•day) | 69.7 (31.4) | 59 (30.1) | 18.1 |
| **140** |  | 329 (25.8) | 278 (26.3) | 18.3 |
| **400** |  | 1140 (21.7) | 983 (21.8) | 16.0 |
| **800** |  | 2400 (20.8) | 2070 (20.8) | 15.9 |
| **50** | C_max_  (µg/mL) | 17.6 (20.0) | 16.7 (20.5) | 5.4 |
| **140** |  | 49.4 (20.0) | 46.7 (20.5) | 5.8 |
| **400** |  | 141 (20.0) | 134 (20.4) | 5.2 |
| **800** |  | 283 (20.0) | 267 (20.4) | 6.0 |

Population PK parameters were calculated based on model predictions from 14 subjects in this Japanese study (GCT1046-02), and 368 subjects from non-Japanese studies GCT1046-01 and GCT1-046-04.

**^a^**Data presented are geometric means (CV%).

Geometric CV% = coefficient of variation = sqrt (exp[variance for log-transformed data] - 1) × 100.

*AUC* area under the concentration-time curve; *C_max_* maximum drug concentration after a single dose; *CV* coefficient of variation; *PK* pharmacokinetic.
